# Comparison of Physical Fitness Profiles Obtained before and during COVID-19 Pandemic in Two Independent Large Samples of Children and Adolescents: DAFIS Project

**DOI:** 10.3390/ijerph19073963

**Published:** 2022-03-26

**Authors:** María Rúa-Alonso, Jessica Rial-Vázquez, Iván Nine, Jose Ramón Lete-Lasa, Iván Clavel, Manuel A. Giráldez-García, Miguel Rodríguez-Corral, Xurxo Dopico-Calvo, Eliseo Iglesias-Soler

**Affiliations:** 1 Performance and Health Group, Faculty of Sports Sciences and Physical Education, Department of Physical Education and Sports, University of A Coruna, 15179 A Coruña, Spain; maria.rua@udc.es (M.R.-A.); jessica.rial@udc.es (J.R.-V.); i.nine@udc.es (I.N.); manuel.avelino.giraldez.garcia@udc.es (M.A.G.-G.); xurxo.dopico@udc.es (X.D.-C.); 2 General Sport Secretariat, Galician Government, 15707 Santiago de Compostela, Spain; jose.ramon.lete.lasa@xunta.gal; 3 Galician Sport Foundation, General Sport Secretariat, Galician Government, 15707 Santiago de Compostela, Spain; ivan.clavel.sanemeterio@xunta.gal (I.C.); miguel.rodriguez.corral@xunta.gal (M.R.-C.)

**Keywords:** schoolchildren, youth population, SARS-CoV-2, lockdown, confinement, anthropometry, muscle strength, agility, flexibility, cardiorespiratory fitness

## Abstract

COVID-19 pandemic restrictions might have negatively affected the health-related physical fitness of children and adolescents. The aim of this study was to contrast the body composition and physical fitness data of two independent samples of children and adolescents obtained from an online database (DAFIS project) before (n = 15,287) and during (n = 2101) the first academic year of the COVID-19 pandemic. The results revealed higher values for the body mass index (*p* = 0.002), waist circumference (*p* < 0.001), and waist to hip and waist to height ratios (*p* < 0.001) during than before the pandemic, particularly in the case of boys. On the other hand, lower muscular fitness was observed for girls during the pandemic. Quantitative and qualitative analysis did not detect relevant changes in cardiorespiratory fitness in children or adolescents (*p* > 0.05). Our data suggested that pandemic constraints might have affected body composition and muscular fitness of children and adolescents. These results might be of interest for designing specific interventions oriented toward counteracting the negative effects of pandemic restrictions on health-related physical fitness.

## 1. Introduction

Physical fitness measurement has emerged as an index of health status in children and youth [1]. Regular physical fitness evaluation allow monitoring it over time and identifying trends in different population groups [2,3,4,5,6,7,8,9,10,11]. To this end, the DAFIS project, integrated in the Plan Galicia Saudable (Healthy Galicia Plan) of the regional government of Galicia (Spain), includes online software that allows the evaluation of the health-related physical fitness of Galician schoolchildren [12].

The COVID-19 pandemic caused by the SARS-CoV-2 virus and the subsequent preventive actions to reduce the number of contagions (social distancing, lockdown, quarantines, lockout, etc.) modified the habits of the population [13,14,15,16,17,18,19] and caused different physical, psychological, and social effects [20,21,22,23,24,25]. In the childhood and youth population, which already exhibited physical activity values below the general recommendations ahead of the pandemic [26,27], these events and policies further reduced physical activity levels and increased sedentary behaviour [28]. With the change from traditional face-to-face teaching to the online modality, the motor engagement developed in physical education classes was reduced to zero, with a consequent drop in physical activity [29]. Moreover, some extracurricular physical and sports practices were called off or limited during the first pandemic year, reducing even further the opportunities for physical exercise in children and adolescents [30]. In addition, dietary habits changed during this time, with generally increased intake and particularly higher consumption of ultra-processed products [14,19,31].

Thus, it can be expected that the physical fitness and body composition of children and adolescents may have been negatively affected by the pandemic constraints, with a consequent deleterious effect on health status. It is therefore necessary to develop studies in order to estimate the impact of the pandemic restrictions on the health-related physical fitness of this population. The DAFIS project can provide relevant information about changes in physical fitness profiles during this period. Thus, the aim of this study was to contrast the body composition and physical fitness data of two independent samples of children and adolescents recorded in DAFIS before and during the first academic year of the COVID-19 pandemic.

## 2. Materials and Methods

Health-related physical fitness of Galician children and adolescents was evaluated using the DAFIS tool (Assessment of physical fitness data; https://dafis.xunta.es; accesesed date: 6 March 2022). Each physical education teacher, who was previously instructed on how to use the software and the application of the physical fitness protocols, conducts evaluations of their students during classes as part of the academic curriculum of the physical education subject. Data from these evaluations are uploaded to the DAFIS tool. From the data uploaded to the online platform, the software provides several health-related physical fitness reports for teachers and families.

A cross-sectional design was used in order to contrast health-related physical fitness data of two samples of children and adolescents, evaluated before and during pandemic. Data were collected from two different periods: prepandemic (from 2012 to March 2020) and pandemic (from October 2020 to June 2021, corresponding to the first academic year after COVID-19 lockdown).

The procedures were in accordance with the Declaration of Helsinki. Furthermore, this study did not require an ethical committee approval, because the collected data corresponded to an institutional project (Galicia Regional Government) that was set up backed by a legal report. Only the information from students whose parents or legal guardians had signed written informed consents were stored in DAFIS. Moreover, to avoid the release of personal information, participants’ names were digitally coded.

### 2.1. Participants

**Figure 1:**
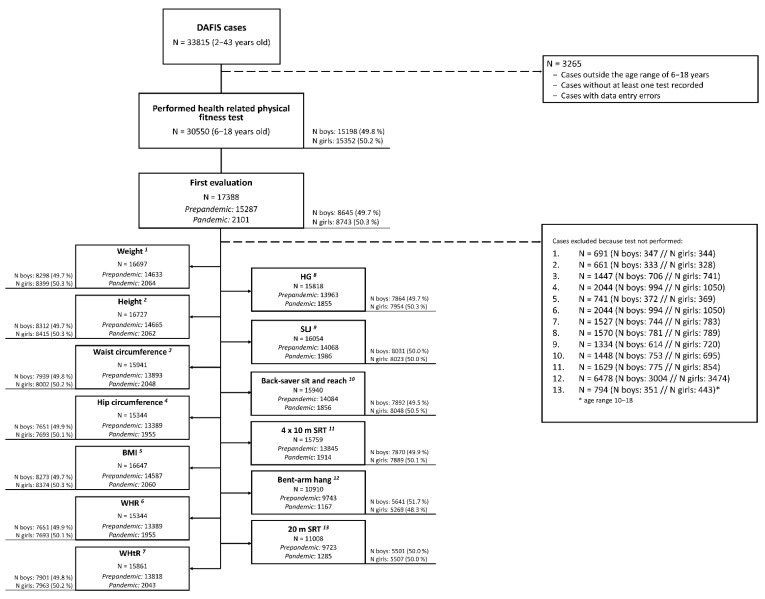
Flowchart of the exclusion criteria. BMI: body mass index; WHR: waist to hip ratio; WHtR: waist to height ratio; HG: handgrip; SLJ: standing long jump; 4 × 10 m SRT: 4 × 10 m shuttle run test; 20 m SRT: 20 m shuttle run test.

A sample of 33,815 cases was extracted from the DAFIS database. Data were removed if the following exclusion criteria were presented: (a) cases with data entry errors; (b) cases outside the age range of 6–18 years; (c) cases without at least one test recorded. After filtering, 17,015 cases (8266 males and 8749 females) were included and considered for the statistical analysis. Of these, 15,287 cases corresponded to the prepandemic period (from 2012 to March 2020) and 2101 to the pandemic period (from October 2020 to June 2021). All cases corresponded to the first evaluation of each participant, i.e., samples from prepandemic and pandemic period were independent (Figure 1).

### 2.2. Anthropometric and Physical Fitness Evaluation: DAFIS Battery

This battery included a total of 10 evaluations. First, four anthropometric measurements, weight, height, and waist and hip circumference, were performed. Body mass index (BMI), waist to hip ratio (WHR) and waist to height ratio (WHtR) were calculated as follows: BMI = weight/height^2^ (kg/m^2^); WHR = Waist circumference/Hip circumference; WHtR = waist circumference/height, respectively.

**Figure 2:**
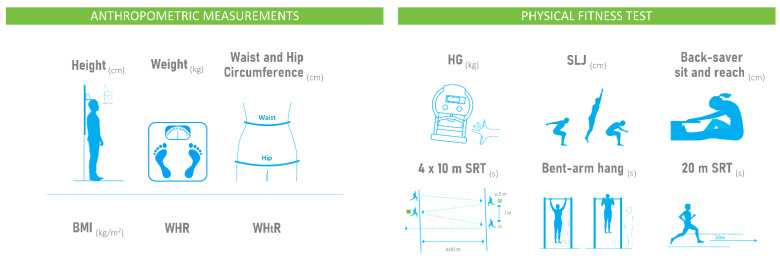
Anthropometric and physical fitness evaluation: DAFIS battery. BMI: body mass index; WHR: waist to hip ratio; WHtR: waist to height ratio; HG: handgrip; SLJ: standing long jump; 4 × 10 m SRT: 4 × 10 m shuttle run test; 20 m SRT: 20 m shuttle run test.

Moreover, 6 physical fitness tests, handgrip strength (HG), standing long jump (SLJ), back-saver sit and reach, 4 × 10 m shuttle run test (4 × 10 m SRT), bent-arm hang, and 20 m shuttle run test (20 m SRT), were completed. Upper and lower body muscular fitness were evaluated by HG, bent-arm hang, and SLJ, whereas back-saver sit and reach was used for a flexibility assessment. Speed–agility and cardiorespiratory fitness were evaluated by the 4 × 10 m SRT and 20 m SRT, respectively. Descriptions of all the procedures have been previously published [12] (Figure 2).

### 2.3. Statistical Analysis

Descriptive values are presented as mean ± SD for quantitative variables and percentages for categorical ones. Pearson’s chi-squared (χ^2^) was used for analysing the association between the samples assessed (prepandemic (cases from academic years before the pandemic) vs. pandemic (cases from the 2020–2021 academic year after lockdown)) and the distributions regarding sex (boys and girls), age groups (6–8 years, 8–10 years, 10–12 years, 12–14 years, 14–16 years, and 16–18 years), and allocation of students in categories of body composition or physical fitness. In this regard, BMI results was categorized (underweight, normal weight, overweight, and obese) according to cut-points previously published [32]. For WHtR, a cut point of 0.5 was considered, as previously suggested [33]. For HG and SLJ, cut points for identifying cardiovascular risk were used [34]. When a significant association was detected, it was interpreted considering both standardized residuals (residuals with absolute values greater than 2 were deemed to be significant). Results from physical fitness tests were analysed by factorial ANOVA with three factors: sample (prepandemic and pandemic), sex (boys and girls), and age group (6–8 years, 8–10 years, 10–12 years, 12–14 years, 14–16 years, and 16–18 years). We focused the analysis in this study on the main effect of sample and its interactions with age group (sample × age), sex (sample × sex), and both age and sex (sample × age × sex). A post hoc *t*-test was carried out with Bonferroni’s adjustment after detecting significant interactions. The effect sizes for main effects and interactions of ANOVA are reported using the partial eta squared (η^2^), whereas for significant pairwise simple contrasts derived from sample × age × sex interaction, Cohen’s d and the corresponding 95% confidence interval (95% CI) are reported. IBM SPSS v.27.0 (IBM Corp., Armonk, NY, USA), GraphPad Prism v.9 (GraphPad Software, San Diego, CA, USA), and Comprehensive Meta-Analysis v.2 (Biostat Inc., Englewood, NJ, USA) were used for statistical analysis, and the statistical significance level was set at 0.05.

## 3. Results

### 3.1. Sex and Age Characteristics of the Samples

A significant association was detected between sample and sex ($\chi_{1}^{2}$ = 7.140; *p* = 0.008). Of the data obtained before the pandemic, 49.3% corresponded to boys and 50.7% to girls, whereas for the data recorded during the pandemic, these percentages were 52.5% and 47.5%, respectively. Nevertheless, differences were small, since the standardized residuals were lower than 2 (Table S1).

Similarly, a significant association was detected between sample and group age ($\chi_{5}^{2}$ = 32.197; *p* < 0.001). Absolute values of standardized residuals were higher than 2 only for the 6–8 year group (14.1% and 17.5% before and during pandemic, respectively; standardized residuals 3.5) and 8–10 year group (17.9% and 15.0% before and in pandemic, respectively; standardized residuals 2.7). The rest of the categories represented similar proportions in both assessments (Table S2).

### 3.2. Health-Related Tests Results

Descriptive values and ANOVA results for body composition are shown in Table 1.

**Figure 3:**
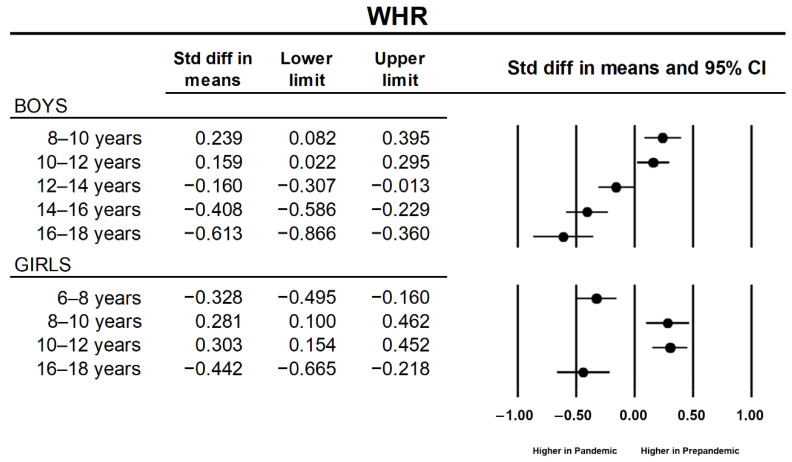
Standardized mean differences (Cohen’s d) between prepandemic and pandemic samples of significant simple pairwise contrasts for waist to hip ratio (WHR).

Regarding BMI, a main effect of sample factor was detected (*p* = 0.002; η^2^ = 0.001) showing higher values for the sample evaluated during the pandemic. A main effect of sample was also observed for waist perimeter (*p* < 0.001; η^2^ = 0.002), with higher values obtained for pandemic assessments. Furthermore, a sample × sex interaction (*p* = 0.012) was also detected. Post hoc analysis showed that values obtained during the pandemic year were significantly higher in comparison with prepandemic years both for boys (*p* < 0.001) and girls (*p* = 0.015). Marginal means increased by 2.9% in boys (70.4 to 72.4 cm) and 1.23% in girls (67.6 to 68.5 cm). For WHR, a significant main effect of sample (*p* < 0.001; η^2^ = 0.001) and sample × age (*p* < 0.001; η^2^ = 0.006) and sample × age × sex (*p* = 0.018; η^2^ = 0.001) interactions were revealed. Post hoc analysis for the sample × age interaction showed higher values for the pandemic year in four age categories (*p* = 0.006, 0.020, <0.001, and <0.001 for the categories of 6–8 years, 12–14 years, 14–16 years, and 16–18 years, respectively) and lower values for the categories 8–10 years and 10–12 years (*p* < 0.001, in both cases). Post hoc simple pairwise comparisons detected an increase in values obtained during the pandemic year for boys in the categories of 12–14 years (*p* = 0.019), 14–16 years (*p* < 0.001), and 16–18 years (*p* < 0.001), whereas these increases were observed in girls only for the categories of 6–8 years (*p* < 0.001) and 16–18 years (*p* < 0.001). A decrease in WHR was observed in both sexes for the categories of 8–10 years (*p* = 0.008 and *p* = 0.004 for boys and girls, respectively) and 10–12 years (*p* = 0.018 and *p* < 0.001 for boys and girls, respectively). Cohen´s d and the corresponding 95% CI are shown in Figure 3. Finally, a significant main effect of sample (*p* < 0.001; η^2^ = 0.002) and a sample × sex interaction (*p* = 0.008; η^2^ < 0.001) were detected for WHtR. Post hoc analysis showed that WHtR was higher in the pandemic year than in prepandemic years (*p* < 0.001) in boys but not in girls (*p* = 0.109).

Descriptive and ANOVA results for physical performance are reported in Table 2.

**Figure 4:**
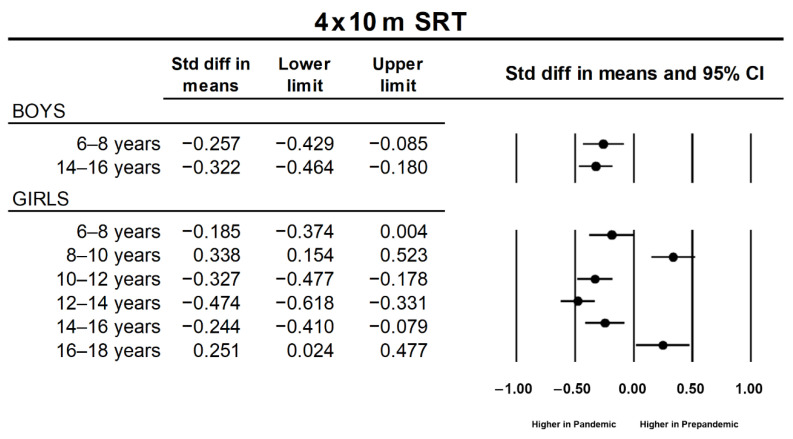
Standardized mean differences (Cohen’s d) between prepandemic and pandemic samples of significant simple pairwise contrasts for 4 × 10 m shuttle run test (4 × 10 m SRT).

Regarding HG test, a significant effect of sample (*p* < 0.001; η^2^ = 0.002) and a sample × age interaction (*p* = 0.033; η^2^ = 0.001) were revealed. Post hoc contrasts showed that for all the age groups, the data obtained during the pandemic year were higher than those obtained during the prepandemic period, although these comparisons were significant only for the 6–8 year, 8–10 year, and 14–16 year age groups (*p* < 0.001 in all the cases). Similarly, a sample × age interaction (*p* = 0.007; η^2^ = 0.001) was observed for SLJ. Post hoc contrasts showed lower performance for pandemic records only for the 10–12 year (*p* = 0.037) and 12–14 year (*p* < 0.001) age groups. As a whole, back-saver sit and reach results were better during the pandemic year than during the prepandemic years (main effect of sample: *p* < 0.001; η^2^ = 0.001). Furthermore, a sample × age interaction (*p* = 0.032; η^2^ = 0.001) was detected, with higher results obtained during the pandemic year for 6–8 year old (*p* = 0.012), 10–12 year old (*p* = 0.002), and 14–16 year old (*p* < 0.001) students. Poorer performance in the 4 × 10 m SRT was observed for pandemic than for prepandemic records (main effect of sample: *p* = 0.023; η^2^ = 0.001). This tendency depended on the age group (sample × age interaction: *p* < 0.001; η^2^ = 0.005), with pandemic results being worse in the 6–8 year (*p* < 0.001), 10–12 year (*p* = 0.016) and 12–14 year (*p* < 0.001) age groups and better in the 8–10 year (*p* < 0.010) and 16–18 year (*p* = 0.010) age groups. On the other hand, a sample × age × sex (*p* < 0.001; η^2^ = 0.00) interaction was detected for the 4 × 10 m SRT. Post hoc pairwise contrasts revealed worse performance during the pandemic year for two categories in boys (6–8 years and 12–14 years; *p* < 0.001 in both cases) and four in girls (6–8 years, 10–12 years, 12–14 years, and 14–16 years; *p* = 0.025, *p* < 0.001, *p* < 0.001, *p* = 0.007, respectively). In contrast, pandemic results were better for girls of 10–12 years (*p* < 0.001) and 16–18 years (*p* = 0.025). The effect sizes for these contrasts are presented in Figure 4.

Significant sample × age interaction (*p* = 0.009; η^2^ = 0.0001) was obtained for the bent-arm hang test. Post hoc analysis did not detect significant differences between samples except for the 16–18 year age group, with higher values recorded during the pandemic year (*p* = 0.010). Finally, a sample × age interaction (*p* < 0.001; η^2^ = 0.003) was significant for the 20 m SRT, with higher values (i.e., better performance) for the 10–12 year age group and lower values for the 12–14 year age group (*p* < 0.001 in both cases) during the pandemic in comparison with the prepandemic period.

### 3.3. Prevalence of Body Composition and Fitness Categories

**Figure 5:**
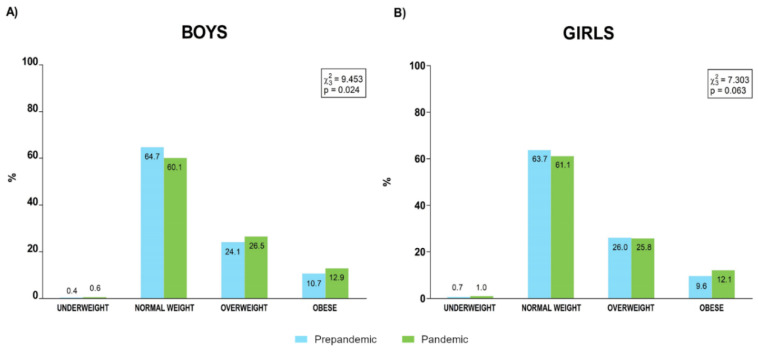
Prevalence of body mass index categories for boys (**A**) and girls (**B**) before and during the pandemic.

Prevalence of categories of BMI are presented in Figure 5.

**Figure 6:**
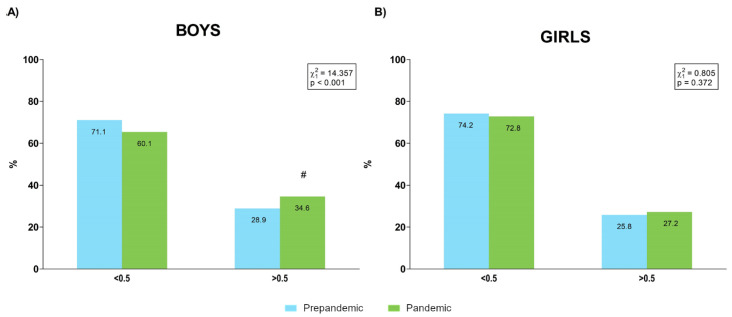
Percentage of boys (**A**) and girls (**B**) over and under the 0.5 cut point for waist to height ratio (WHtR) before and during the pandemic. #: absolute value of the standardized residual ≥ 2 for this category.

The percentages of samples over and under the 0.5 WHtR cut point for each period are shown in Figure 6.

**Figure 7:**
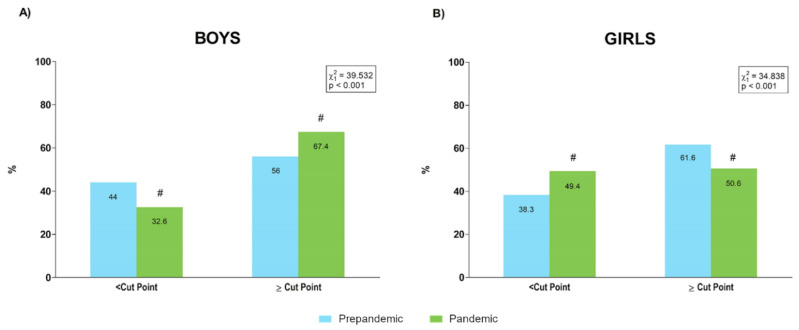
Percentages of boys (**A**) and girls (**B**) over and under cardiovascular risk handgrip performance cut points [34] before and during the pandemic. #: absolute value of the standardized residual ≥ 2 for this category.

The proportions of boys and girls over and under cardiovascular risk HG cut points [34] are presented in Figure 7. These values corresponded to boys of 6 to 10 and 12 to 16 years, since the reference values that we used were derived for these age groups.

**Figure 8:**
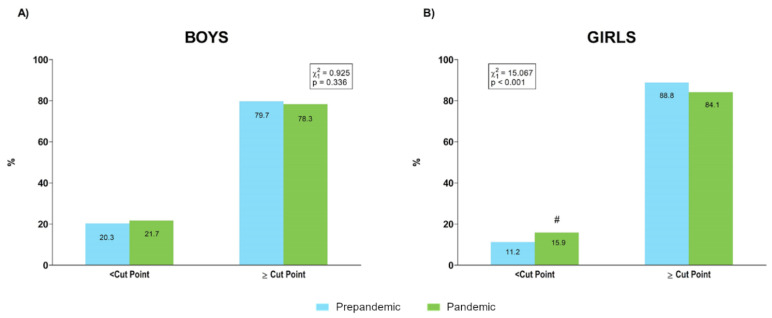
Percentage of boys (**A**) and girls (**B**) over and under cardiovascular risk standing long jump performance cut points [34] before and during the pandemic. #: absolute value of the standardized residual ≥ 2 for this category.

The percentages of boys and girls over and under cardiovascular risk relative SLJ cut points that have been previously identified [34] are shown in Figure 8. As pointed out for HG, these values were established only for boys and girls of 6 to 10 and 12 to 16 years.

**Figure 9:**
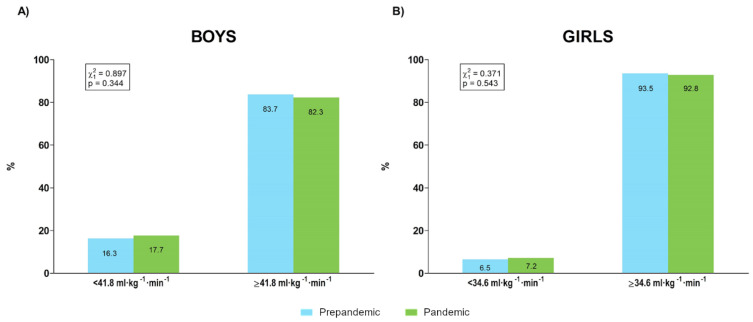
Percentages of boys (**A**) and girls (**B**) over and under cardiovascular risk 20 m shuttle run test performance cut points [35] before and during the pandemic.

The proportions of boys and girls over and under cardiovascular risk 20 m SRT performance cut points [35] for samples evaluated before and during a pandemic academic year are represented in Figure 9.

All contingency tables with the distributions of prepandemic and pandemic samples for body composition (i.e., BMI and WHtR) and physical fitness (i.e., HG, SLJ and 20 m SRT) categories are provided in the Supplementary Material for both boys and girls (Tables S3–S12).

## 4. Discussion

The present study provides information about the changes during the pandemic in the body composition and physical fitness profiles of children and adolescents evaluated in school centres following a standardized protocol. Our results reflected changes in body composition particularly in the male population, whereas muscular performance was reduced especially in girls. In contrast, quantitative and qualitative analysis did not detect relevant changes in cardiorespiratory fitness in the school population.

Several results of our analysis pointed to less healthy body composition in the sample evaluated during the pandemic year, although this tendency was clearer in boys than in girls. Quantitative analysis revealed a global increase in BMI, while the prevalence of overweight and obese categories increased in the pandemic year in both boys and girls, although this change in the distribution was significant only for boys. While WHR showed similar behaviour in both sexes, significant increases in WHtR values and the prevalence of population over the 0.5 cut point were detected in boys but not in girls. Specifically, 28.9% of boys were over the cut point prepandemic, and this value increased to 34.6% in pandemic, whereas girls presented similar values prepandemic (25.8%) and during the pandemic (27.2%) (Figure 6). COVID-19 lockdown and subsequent restriction have had a relevant effect on habits [36,37] and physical activity levels [28,38], influencing the weight gain and adiposity increases detected in this population during pandemic [25,36,39]. Although it has been reported that upon return to school after lockdown, levels of physical activity increased in both sexes but especially in boys [38], our data would suggest that this increase in physical activity would not be sufficient to fully counteract the effect of some sedentary habits on the body composition of the male school population. Unfortunately, the DAFIS platform does not include physical activity or habit data, which would have been greatly useful in order to identify the causality of this tendency.

In contrast to body composition, lower levels of muscular fitness were observed mainly in the female sample during the pandemic year. Thus, whereas the prevalence of boys over HG healthy cut points was higher during the pandemic (prepandemic: 56% and pandemic: 67.4%), this percentage was lower for girls during than before the pandemic (prepandemic: 61.6% and pandemic: 50.6%), suggesting lower and higher prevalence of cardiovascular risk profiles for boys and girls, respectively, who were evaluated during the pandemic year in comparison than in those evaluated during the prepandemic period (Figure 7). Furthermore, the prevalence of SLJ results under healthy cut points increased during the pandemic only for girls, while a worse 4 × 10 m SRT performance was detected during the pandemic in four out of six age groups for girls but in only two out of six age categories in boys. Recently, a study showed that during lockdown, levels of physical activity were similar between boys and girls, whereas traditional differences between sexes (i.e., girls engaged in less moderate-to vigorous physical activity than boys) in physical activity were re-established on return to school [38]. Thus, it can be speculated that physical activity resumed by girls after lockdown would have been insufficient for maintaining proper stimulation of the muscular component. Given the relevance of muscular fitness to the health status of children and adolescents [40,41], our results would suggest the implementation of specific programs for improving muscular fitness in the school population, and specifically in girls, to avoid the deleterious effect of pandemic restrictions on this component.

Unlike muscular fitness, both quantitative and qualitative analysis reflected that cardiorespiratory fitness was similar between prepandemic and pandemic measurements. A previously published principal component analysis of prepandemic DAFIS data [12] showed that body composition and muscular fitness accumulated most of their variability in the health-related fitness of children and adolescents. Therefore, it might be possible that cardiorespiratory fitness was less sensitive for detecting differences in physical fitness between the prepandemic and pandemic samples. On the other hand, pandemic restrictions could have mainly limited opportunities for practicing the vigorous physical activity required for muscular stimulation but less constrained opportunities for developing moderate physical activity that would allow partially preserving cardiorespiratory fitness. On the other hand, it must be pointed out that during the pandemic year, the 20 m SRT was performed while students wore face masks, and therefore, these results would support the lack of influence of this condition on maximum cardiorespiratory performance [42].

There are some limitations in this study that must be considered. First, the sampling procedure was not probabilistic, although the sample sizes could have partially counteracted possible bias risk. Second, there were some differences in sample composition regarding age groups and sex distribution, although these deviations were small. Third, the physical activity levels of each cohort were not available for analysis. Finally, since this was not a repeated measures design, the effect of pandemic on physical fitness was only indirectly estimated.

## 5. Conclusions

In conclusion, our results showed that the body composition of boys evaluated during the first pandemic year was worse than that of their counterparts assessed in prepandemic years. Additionally, girls evaluated during the pandemic showed lower muscular fitness than those assessed during the prepandemic period. These results might be of interest for designing specific interventions oriented toward counteracting the negative effects of pandemic restrictions on health-related fitness.

## Figures and Tables

**Table 1:** Body composition values before and during pandemic.

			Age Group						ANOVA *p*-Value (η^2^)						
Test	Sex	Sample	6–8 Years	8–10 Years	10–12 Years	12–14 Years	14–16 Years	16–18 Years	S	A	Sample	S × A	S × Sample	A × Sample	S × A × Sample
**Waist Circumference (cm)**	Boys	Pre	59.77 ± 6.76	64.68 ± 8.99	69.04 ± 10.04	73.48 ± 11.51	76.66 ± 10.74	78.62 ± 10.51	<0.001 (0.012)	<0.001 (0.129)	<0.001 (0.002)	<0.001 (0.006)	0.012 (0.000398)	0.128 (0.000537)	0.092 (0.000594)
		Post	60.56 ± 7.57	66.21 ± 8.90	70.71 ± 10.23	75.01 ± 12.32	80.95 ± 11.34	81.14 ± 13.47							
	Girls	Pre	59.10 ± 6.58	64.25 ± 8.58	67.48 ± 9.49	70.72 ± 10.18	71.85 ± 9.72	72.47 ± 9.61							
		Post	60.52 ± 7.53	64.9 ± 10.06	67.00 ± 9.08	72.6 ± 10.67	72.91 ± 10.01	72.97 ± 8.83							
**Hip Circumference (cm)**	Boys	Pre	68.51 ± 6.11	74.86 ± 8.19	80.53 ± 8.76	86.28 ± 9.82	92.06 ± 9.69	95.60 ± 9.30	0.102 (0.000174)	<0.001 (0.288)	<0.001 (0.001)	<0.001 (0.002)	0.223 (0.000097)	<0.001 (0.002)	0.115 (0.00057)
		Post	68.63 ± 6.81	77.38 ± 8.20	84.29 ± 8.74	86.98 ± 11.44	94.97 ± 10.61	92.49 ± 10.92							
	Girls	Pre	68.71 ± 6.28	75.21 ± 7.95	81.38 ± 9.59	88.04 ± 9.99	93.34 ± 9.38	95.12 ± 9.67							
		Post	68.11 ± 7.22	77.74 ± 9.53	83.47 ± 10.07	90.72 ± 10.62	92.69 ± 10.56	92.80 ± 12.12							
**BMI (kg/m^2^)**	Boys	Pre	17.33 ± 2.74	18.85 ± 3.38	19.81 ± 3.61	21.15 ± 4.09	21.99 ± 4.11	22.93 ± 3.96	0.014 (0.000363)	<0.001 (0.095)	0.002 (0.001)	0.001 (0.001)	0.440 (0.000036)	0.651 (0.000200)	0.117 (0.001)
		Post	17.63 ± 2.71	19.42 ± 3.64	20.18 ± 3.93	21.05 ± 4.42	22.58 ± 4.19	22.51 ± 4.36							
	Girls	Pre	17.32 ± 2.60	18.77 ± 3.19	19.82 ± 3.79	21.42 ± 4.03	22.67 ± 4.07	22.99 ± 4.33							
		Post	17.73 ± 2.96	19.08 ± 3.71	19.62 ± 3.66	22.05 ± 4.30	23.12 ± 4.42	23.56 ± 4.18							
**WHR**	Boys	Pre	0.88 ± 0.05	0.87 ± 0.06	0.86 ± 0.07	0.85 ± 0.07	0.83 ± 0.07	0.82 ± 0.07	<0.001 (0.031)	<0.001 (0.049)	<0.001 (0.001)	<0.001 (0.014)	0.057 (0.000237)	<0.001 (0.006)	0.018 (0.001)
		Post	0.88 ± 0.07	0.85 ± 0.06	0.85 ± 0.07	0.86 ± 0.10	0.86 ± 0.08	0.86 ± 0.08							
	Girls	Pre	0.87 ± 0.06	0.86 ± 0.06	0.83 ± 0.07	0.80 ± 0.07	0.77 ± 0.07	0.76 ± 0.06							
		Post	0.89 ± 0.08	0.84 ± 0.08	0.81 ± 0.08	0.81 ± 0.10	0.78 ± 0.07	0.79 ± 0.07							
**WHtR**	Boys	Pre	0.48 ± 0.05	0.48 ± 0.06	0.47 ± 0.06	0.47 ± 0.07	0.45 ± 0.06	0.45 ± 0.06	<0.001 (0.003)	<0.001 (0.015)	<0.001 (0.002)	<0.001 (0.002)	0.008 (0.000449)	0.322 (0.000369)	0.194 (<0.001)
		Post	0.49 ± 0.05	0.49 ± 0.06	0.49 ± 0.06	0.48 ± 0.07	0.47 ± 0.06	0.47 ± 0.08							
	Girls	Pre	0.48 ± 0.05	0.48 ± 0.06	0.46 ± 0.06	0.45 ± 0.06	0.45 ± 0.06	0.45 ± 0.06							
		Post	0.49 ± 0.05	0.48 ± 0.07	0.46 ± 0.06	0.46 ± 0.06	0.45 ± 0.06	0.45 ± 0.05							

BMI: body mass index; WHR: waist to hip ratio; WHtR: waist to height ratio; Pre: Prepandemic; Post: Pandemic; S: sex; A: age.

**Table 2:** Physical fitness values before and during pandemic.

			Age Group						ANOVA *p*-Value (η^2^)						
Test	Sex	Sample	6–8 Years	8–10 Years	10–12 Years	12–14 Years	14–16 Years	16–18 Years	S	A	Sample	S × A	S × Sample	A × Sample	S × A × Sample
**Handgrip (kg)**	Boys	Pre	19.87 ± 4.48	26.65 ± 5.95	34.11 ± 7.86	45.99 ± 12.30	64.21 ± 15.20	74.16 ± 15.01	<0.001 (0.060)	<0.001 (0.472)	<0.001 (0.002)	<0.00 (0.068)	0.157 (0.0001)	0.033 (0.001)	0.200 (0.0001)
		Post	22.47 ± 4.14	28.33 ± 6.48	34.89 ± 7.44	45.05 ± 11.47	66.44 ± 17.78	74.12 ± 15.73							
	Girls	Pre	18.43 ± 4.27	25.26 ± 5.61	33.39 ± 7.97	42.34 ± 9.01	47.95 ± 8.79	49.32 ± 9.06							
		Post	20.53 ± 4.33	27.65 ± 5.87	33.88 ± 8.44	43.84 ± 9.02	49.69 ± 10.22	51.54 ± 8.58							
**SLJ (cm)**	Boys	Pre	107.81 ± 19.41	123.88 ± 21.35	139.91 ± 23.37	157.23 ± 26.95	182.10 ± 29.29	197.10 ± 29.87	<0.001 (0.060)	<0.001 (0.261)	0.187 (0.0001)	<0.001 (0.031)	0.801 (0.0001)	0.007 (0.001)	0.455 (0.0001)
		Post	108.68 ± 19.69	124.70 ± 21.21	138.39 ± 25.62	154.60 ± 28.93	182.39 ± 31.10	193.55 ± 28.15							
	Girls	Pre	100.09 ± 17.70	117.06 ± 19.47	133.46 ± 22.40	142.93 ± 23.21	148.23 ± 24.82	148.61 ± 24.78							
		Post	99.57 ± 19.01	120.50 ± 24.37	129.86 ± 20.56	137.23 ± 25.34	150.05 ± 27.32	149.30 ± 25.59							
**Back-saver sit and reach (cm)**	Boys	Pre	24.21 ± 5.68	22.53 ± 5.99	22.07 ± 6.16	21.39 ± 6.37	23.56 ± 7.60	25.50 ± 7.89	<0.001 (0.052)	<0.001 (0.016)	<0.001 (0.001)	<0.001 (0.005)	0.176 (0.0001)	0.032 (0.001)	0.837 (0.0001)
		Post	25.49 ± 5.43	22.68 ± 6.39	22.84 ± 6.74	21.79 ± 7.63	25.28 ± 9.17	24.89 ± 9.10							
	Girls	Pre	27.21 ± 5.77	26.53 ± 6.45	26.42 ± 7.00	28.11 ± 7.39	30.51 ± 8.09	30.74 ± 7.73							
		Post	28.19 ± 4.92	27.44 ± 6.87	27.97 ± 7.09	28.45 ± 7.68	32.65 ± 8.80	31.45 ± 8.43							
**4 × 10 m SRT (s)**	Boys	Pre	15.60 ± 2.05	14.46 ± 1.83	13.51 ± 1.53	12.50 ± 1.43	11.68 ± 1.31	11.26 ± 1.84	<0.001 (0.028)	<0.001 (0.206)	0.023 (0.0001)	<0.001 (0.009)	0.119 (0.0001)	<0.001 (0.005)	<0.001 (0.002)
		Post	16.18 ± 3.02	14.29 ± 2.08	13.39 ± 1.70	12.97 ± 1.73	11.44 ± 2.09	10.94 ± 1.63							
	Girls	Pre	16.19 ± 1.85	14.89 ± 1.63	13.85 ± 1.54	13.14 ± 1.35	12.94 ± 1.38	12.91 ± 1.42							
		Post	16.54 ± 2.19	14.34 ± 1.65	14.38 ± 1.99	13.78 ± 1.45	13.31 ± 2.10	12.53 ± 1.95							
**Bent-Arm Hang (s)**	Boys	Pre	6.06 ± 5.81	8.75 ± 8.65	11.09 ± 10.81	13.41 ± 13.41	23.44 ± 17.60	28.40 ± 19.05	<0.001 (0.028)	<0.001 (0.065)	0.868 (0.0001)	<0.001 (0.023)	0.648 (0.0001)	0.009 (0.0001)	0.094 (0.001)
		Post	5.54 ± 5.42	8.80 ± 8.41	8.34 ± 10.03	13.50 ± 12.76	27.58 ± 25.18	29.99 ± 20.34							
	Girls	Pre	5.26 ± 5.10	6.57 ± 7.06	7.40 ± 8.09	9.22 ± 10.20	10.29 ± 11.84	11.78 ± 13.25							
		Post	3.81 ± 3.38	8.46 ± 7.94	7.25 ± 8.36	6.39 ± 6.53	10.78 ± 13.46	13.15 ± 15.47							
**20 m SRT (stages)**	Boys	Pre			4.26 ± 2.34	5.28 ± 2.62	6.58 ± 2.89	7.21 ± 3.00	<0.001 (0.061)	<0.001 (0.029)	0.951 (0.0001)	<0.001 (0.010)	0.960 (0.0001)	<0.001 (0.003)	0.302 (0.0001)
		Post			4.84 ± 3.23	4.68 ± 2.51	6.73 ± 3.07	7.07 ± 3.20							
	Girls	Pre			3.35 ± 1.88	3.77 ± 1.90	3.88 ± 1.90	4.16 ± 1.98							
		Post			3.55 ± 2.44	3.28 ± 1.80	3.89 ± 2.10	4.42 ± 2.37							

HG: handgrip; SLJ: standing long jump; 4 × 10 m SRT: 4 × 10 m shuttle run test; 20 m SRT: 20 m shuttle run test; Pre: Prepandemic; Post: Pandemic; S: sex; A: age.

## Data Availability

The original database belongs to the Galician Regional Government. It can be requested directly from the following e-mail address: ivan.clavel.sanemeterio@xunta.gal.

## Supplementary Materials

The following supporting information can be downloaded at: https://www.mdpi.com/article/10.3390/ijerph19073963/s1, Table S1: Pearson’s chi-squared (χ^2^). Association between the samples assessed (prepandemic and pandemic) and distribution regarding sex; Table S2: Pearson’s chi-squared (χ^2^). Association between the samples assessed (prepandemic and pandemic) and distribution regarding age groups; Table S3: Pearson’s chi-squared (χ^2^). Association between the samples assessed (prepandemic and pandemic) and distribution regarding body mass index category for boys; Table S4: Pearson’s chi-squared (χ^2^). Association between the samples assessed (prepandemic and pandemic) and distribution regarding body mass index category for girls; Table S5: Pearson’s chi-squared (χ^2^). Association between the samples assessed (prepandemic and pandemic) and distribution regarding waist to height ratio cut points for boys; Table S6: Pearson’s chi-squared (χ^2^). Association between the samples assessed (prepandemic and pandemic) and distribution regarding waist to height ratio cut points for girls; Table S7: Pearson’s chi-squared (χ^2^). Association between the samples assessed (prepandemic and pandemic) and distribution regarding handgrip cut points for boys; Table S8: Pearson’s chi-squared (χ^2^). Association between the samples assessed (prepandemic and pandemic) and distribution regarding handgrip cut points for girls; Table S9: Pearson’s chi-squared (χ^2^). Association between the samples assessed (prepandemic and pandemic) and distribution regarding standing long jump cut points for boys; Table S10: Pearson’s chi-squared (χ^2^). Association between the samples assessed (prepandemic and pandemic) and distribution regarding standing long jump cut points for girls; Table S11: Pearson’s chi-squared (χ^2^). Association between the samples assessed (prepandemic and pandemic) and distribution regarding 20 m shuttle run test cut points for boys; Table S12: Pearson’s chi-squared (χ^2^). Association between the samples assessed (prepandemic and pandemic) and distribution regarding 20 m shuttle run test cut points for girls.
