# Supplementary material for: Comparison of Physical Fitness Profiles Obtained before and during COVID-19 Pandemic in Two Independent Large Samples of Children and Adolescents: DAFIS Project
Source: Int J Environ Res Public Health. 2022 Mar 26;19(7):3963. doi: 10.3390/ijerph19073963 (PMC8998010; doi:10.3390/ijerph19073963)
Supplement: Supplementary file 1 [file ijerph-19-03963-s001.zip › ijerph-1619942-supplementary.pdf]

Table S1. Pearson's chi-squared (χ^2). Association between the sample assessed (Prepandemic and Pandemic) and distribution regarding sex.

		SEX		
		**Boys**	**Girls**	**Total**
**Prepandemic**	Count	7543	7744	15287
	Expected Count	7600.4	7686.6	15287.0
	% within Pre-pandemic; Pandemic	49.3%	50.7%	100%
	Standardized Residual	-0.7	0.7	
**Pandemic**	Count	1102	999	2101
	Expected Count	1044.6	1056.4	2101.0
	% wiithin Pre-pandemic; Pandemic	52.5%	47.5%	100%
	Standardized Residual	1.8	-1.8	

χ^2_1= 7.140; p = 0.008

Table S2. Pearson's chi-squared (χ^2). Association between the sample assessed (Prepandemic and Pandemic) and distribution regarding age groups.

		Age group						
		**6-8 years**	**8-10 years**	**10-12 years**	**12-14 years**	**14-16 years**	**16-18 years**	**Total**
**Prepandemic**	Count	2163	2736	3060	3508	2374	1446	15287
	Expected Count	2224.3	2683.2	3088.5	3472.7	2394.9	1423.4	15287.0
	% within Pre-pandemic; Pandemic	14.1%	17.9%	20.0%	22.9%	15.5%	9.5%	100%
	Standardized Residual	-1.3	1.0	-0.5	0.6	-0.4	0.6	
**Pandemic**	Count	367	316	453	442	350	173	2101
	Expected Count	305.7	368.8	424.5	477.3	329.1	195.6	2101.0
	% wiithin Pre-pandemic; Pandemic	17.5%	15.0%	21.6%	21.0%	16.7%	8.2%	100%
	Standardized Residual	3.5	-2.7	1.4	-1.6	1.1	-1.6	

$\chi^2_5 = 32.197$; $p < 0.001$

Table S3. Pearson's chi-squared (χ^2). Association between the sample assessed (Prepandemic and Pandemic) and distribution regarding Body Mass Index category for boys.

Boys		Body Mass Index Category				
		Underweight	Normal Weight	Overweight	Obesity	Total
**Prepandemic**	Count	29	4654	1736	773	7192
	Expected Count	30.4	4610.9	1757.8	792.8	7192.0
	% within Pre-pandemic; Pandemic	0.4%	64.7%	24.1%	10.7%	100%
	Standardized Residual	-0.3	0.6	-0.5	-0.7	
**Pandemic**	Count	6	650	286	139	1081
	Expected Count	4.6	693.1	264.2	119.2	1081.0
	% wiithin Pre-pandemic; Pandemic	0.6%	60.1%	26.5%	12.9%	100%
	Standardized Residual	0.7	-1.6	1.3	1.8	

χ^2_3= 9.453; p = 0.024

Table S4. Pearson's chi-squared (χ^2). Association between the sample assessed (Prepandemic and Pandemic) and distribution regarding Body Mass Index category for girls.

Girls		Body Mass Index Category				
		Underweight	Normal Weight	Overweight	Obesity	Total
**Prepandemic**	Count	52	4709	1923	711	7395
	Expected Count	54.8	4686.6	1921.6	732.1	7395.0
	% within Pre-pandemic; Pandemic	0.7%	63.7%	26.0%	9.6%	100%
	Standardized Residual	-0.4	0.3	0	-0.8	
**Pandemic**	Count	10	598	253	118	979
	Expected Count	7.2	620.4	254.4	96.9	979.0
	% wiithin Pre-pandemic; Pandemic	1.0%	61.1%	25.8%	12.1%	100%
	Standardized Residual	1.0	-0.9	-0.1	2.1	

χ^2_3= 7.303; p = 0.063

Table S5. Pearson's chi-squared (χ^2). Association between the sample assessed (Prepandemic and Pandemic) and distribution regarding Waist to Height Ratio cut points for boys.

		**Waist to Height Ratio**		
**Boys**		**<0.5**	**>0.5**	**Total**
**Prepandemic**	Count	4854	1975	6829
	Expected Count	4801.3	2027.7	6829.0
	% within Pre-pandemic; Pandemic	71.1%	28.9%	100%
	Standardized Residual	0.8	-1.2	
**Pandemic**	Count	701	371	1072
	Expected Count	75.7	318.3	1072.0
	% wiithin Pre-pandemic; Pandemic	65.4%	34.6%	100%
	Standardized Residual	-1.9	3.0	

χ^2_1= 14.357; p <0.001

Table S6. Pearson's chi-squared (χ^2). Association between the sample assessed (Prepandemic and Pandemic) and distribution regarding Waist to Height Ratio cut points for girls.

		**Waist to Height Ratio**		
**Girls**		**<0.5**	**>0.5**	**Total**
**Prepandemic**	Count	5183	1806	6989
	Expected Count	5171.5	1817.5	6989.0
	% within Pre-pandemic; Pandemic	74.2%	25.8%	100%
	Standardized Residual	0.2	-0.3	
**Pandemic**	Count	707	264	971
	Expected Count	718.5	252.5	971.0
	% wiithin Pre-pandemic; Pandemic	72.8%	27.2%	100%
	Standardized Residual	-0.4	0.7	

χ^2_1= 0.805; p = 0.370

Table S7. Pearson's chi-squared (χ^2). Association between the sample assessed (Prepandemic and Pandemic) and distribution regarding Handgrip cut points for boys.

		Handgrip		
**Boys**		**<Cut Point**	**≥Cut Point**	**Total**
**Prepandemic**	Count	3257	2561	5818
	Expected Count	3341.5	2476.5	5818.0
	% within Pre-pandemic; Pandemic	56.0%	44.0%	100%
	Standardized Residual	-1.5	1.7	
**Pandemic**	Count	571	276	847
	Expected Count	486.5	360.5	847.0
	% wiithin Pre-pandemic; Pandemic	67.4%	32.6%	100%
	Standardized Residual	3.8	-4.5	

χ^2_1= 39.532; p <0.001

Table S8. Pearson's chi-squared (χ^2). Association between the sample assessed (Prepandemic and Pandemic) and distribution regarding Handgrip cut points for girls.

		Handgrip		
**Girls**		**<Cut Point**	**≥Cut Point**	**Total**
**Prepandemic**	Count	3707	2303	6010
	Expected Count	3631.9	2378.1	6010.0
	% within Pre-pandemic; Pandemic	61.7%	38.3%	100%
	Standardized Residual	1.2	-1.5	
**Pandemic**	Count	386	377	763
	Expected Count	461.1	301.9	763.0
	% wiithin Pre-pandemic; Pandemic	50.6%	49.4%	100%
	Standardized Residual	-3.5	4.3	

χ^2_1= 34.828; p <0.001

Table S9. Pearson's chi-squared (χ^2). Association between the sample assessed (Prepandemic and Pandemic) and distribution regarding Standing Long Jump cut points for boys.

		Standing Long Jump		
**Boys**		**<Cut Point**	**≥Cut Point**	**Total**
**Prepandemic**	Count	4825	1230	6055
	Expected Count	4814.0	1241,0	6055,0
	% within Pre-pandemic; Pandemic	79.7%	20.3%	100%
	Standardized Residual	0.2	-0.3	
**Pandemic**	Count	726	201	927
	Expected Count	737,0	190,0	927,0
	% wiithin Pre-pandemic; Pandemic	78.3%	21.7%	100%
	Standardized Residual	-0.4	0.8	

$\chi^2_1 = 0.925$; $p = 0.336$

Table S10. Pearson's chi-squared (χ^2). Association between the sample assessed (Prepandemic and Pandemic) and distribution regarding Standing Long Jump cut points for girls.

		Standing Long Jump		
**Girls**		**<Cut Point**	**≥Cut Point**	**Total**
**Prepandemic**	Count	5490	695	6185
	Expected Count	5456.4	728.6	6185.0
	% within Pre-pandemic; Pandemic	88.8%	11.2%	100%
	Standardized Residual	0.5	-1.2	
**Pandemic**	Count	688	130	818
	Expected Count	721.6	96.4	818.0
	% wiithin Pre-pandemic; Pandemic	84.1%	15.9%	100%
	Standardized Residual	-1.3	3.4	

$\chi^2_1 = 15.067$; $p < 0.001$

Table S11. Pearson's chi-squared (χ^2). Association between the sample assessed (Prepandemic and Pandemic) and distribution regarding 20m Shuttle Run Test cut points for boys.

		20m Shuttle Run Test		
**Boys**		**<41.8ml.kg^{-1}.min^{-1}**	**≥41.8ml.kg^{-1}.min^{-1}**	**Total**
**Prepandemic**	Count	4050	787	4837
	Expected Count	4041.5	795.5	4837.0
	% within Pre-pandemic; Pandemic	83.7%	16.3%	100%
	Standardized Residual	0.1	-0.3	
**Pandemic**	Count	543	117	660
	Expected Count	551.5	108.5	660.0
	% wiithin Pre-pandemic; Pandemic	82.3%	17.7%	100%
	Standardized Residual	-0.4	0.8	

χ^2_1= 0.897; p = 0.344

Table S12. Pearson's chi-squared (χ^2). Association between the sample assessed (Prepandemic and Pandemic) and distribution regarding 20m Shuttle Run Test cut points for girls.

		20m Shuttle Run Test		
**Girls**		**<34.6ml.kg^{-1}.min^{-1}**	**≥34.6ml.kg^{-1}.min^{-1}**	**Total**
**Prepandemic**	Count	4568	318	4886
	Expected Count	4564.5	321.5	4886.0
	% within Pre-pandemic; Pandemic	93.5%	6.5%	100%
	Standardized Residual	0.1	-0.2	
**Pandemic**	Count	571	44	615
	Expected Count	574.5	40.5	615.0
	% wiithin Pre-pandemic; Pandemic	92.8%	7.2%	100%
	Standardized Residual	-0.1	0.6	

χ^2_1= 0.371; p = 0.543
